# Dengue and COVID-19 infections in the ASEAN region: a concurrent outbreak of viral diseases

**DOI:** 10.4178/epih.e2021070

**Published:** 2021-09-16

**Authors:** Lowilius Wiyono, Ian Christoper N. Rocha, Trisha Denise D. Cedeño, Adriana Viola Miranda, Don Eliseo Lucero-Prisno III

**Affiliations:** 1 Faculty of Medicine, Universitas Indonesia, Jakarta, Indonesia; 2 School of Medicine, Centro Escolar University, Manila, Philippines; 3 Department of Global Health and Development, London School of Hygiene and Tropical Medicine, London, UK; 4 Faculty of Management and Development Studies, University of the Philippines Open University, Los Baños, Philippines

**Keywords:** Asia, COVID-19, Dengue, Infection control

## Abstract

Member countries of the Association of Southeast Asian Nations (ASEAN) have faced dengue outbreaks for decades, and the region has one of the highest rates of dengue globally. Outbreaks continue to occur concurrently with the coronavirus disease 2019 (COVID-19) pandemic in the 10 ASEAN countries. Both infectious diseases pose a tremendous burden in these countries related to both infection control and the economy. Increases in the number of dengue cases occurred in part due to disruptions in the pathogen-host-vector relationship caused by changes in human behaviour in response to the COVID-19 pandemic. The spread of dengue was further aggravated by the implementation of lockdowns and social distancing policies. These measures limited the coverage of dengue preventive programs and delayed the medical management of both diseases due to co-infection and misdiagnosis. It is of the utmost importance for the population to remain aware of both diseases, and dengue vector control strategies must be devised to properly address outbreaks using digitalization and remote surveillance. Similarly, critical triage algorithms and further research are also needed to combat co-infection and misdiagnosis. Controlling the spread of COVID-19 though vaccination should also be undertaken to reduce the impact of the pandemic.

## INTRODUCTION

The pandemic of coronavirus disease 2019 (COVID-19), which is caused by the severe acute respiratory syndrome coronavirus 2 (SARS-CoV-2) virus, continues to affect populations on a global scale. With an increasing number of COVID-19 cases and the uncontrolled spread of the disease, the countries of the Association of Southeast Asian Nations (ASEAN) constitute one of the world’s most affected regions. The World Health Organization (WHO) reported a 6.2% increase in new COVID-19 cases compared to other regions which have seen a decline in new cases by as much as 5% [1]. As of this study, the ASEAN region now has more than 3 million active COVID-19 cases with an increasing trend in total cases, and a declining trend is not expected in the near future [2]. COVID-19, however, is not the only viral infection threatening the well-being of the ASEAN population. Despite its long history, dengue infection also continues to be one of the major public health issues affecting the region.

Dengue virus is one of the most widespread and rapidly proliferating vector-borne diseases in the world, with more than 390 million infections per year. Dengue is endemic in more than 100 countries [3]. The ASEAN region has seen a significant increase in dengue cases and is considered the global epicentre of dengue infection. The ASEAN region has seen a 46% increase in dengue cases from 2015 to 2019, with Indonesia, Myanmar, and Thailand being some of the most highly endemic countries in the world. The region contributes to more than half of the global burden of dengue [4].

**Figure 1.**
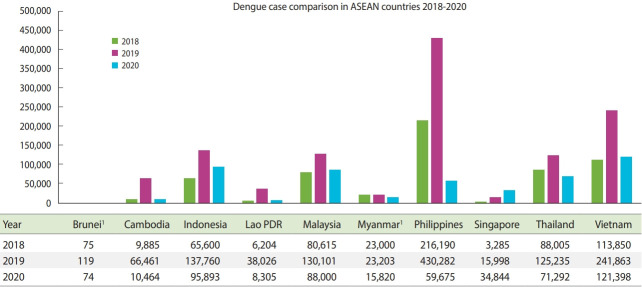
Comparison of dengue cases between 2018 and 2020 in several Associa tion of Southeast Asian Nations (ASEAN) countries; Singapore reported higher cases of dengue in 2020 (during the pandemic), while others have shown decrease as compared to the dengue epidemics in 2019. ^1^Data is extrapol.

The worsening state of the COVID-19 pandemic in the region poses a challenge for authorities who have made substantial efforts throughout the years to mitigate the impact of dengue. Consequently, the number of dengue cases remains high and has continuously increased during the COVID-19 pandemic. Several countries have made similar observations. Singapore reported 1,793 weekly dengue cases during the end of second quarter of 2020 and had a total of more than 38,000 cases in 2020, which surpassed the 2013 national record in the number of annual new dengue cases. Indonesia’s Ministry of Health reported 137,760 new dengue cases in 2019, which was almost twice the number of cases in the previous year. While the disease has a low mortality rate, better therapeutic management could still be implemented to mitigate its emergence and recurrence. Thailand reported more than 40,000 new dengue cases during the second half of the year, with a major surge occurring in the span of months. Similar trends have been observed in the Philippines, where 3 times the number of cases from the same period last year have been reported [5]. Increases in the number of dengue cases are still being observed in various countries, though not to the same degree as the 2019 dengue epidemic (Figure 1). COVID-19 has posed a challenge for controlling dengue infection, which has led to an increased burden of dengue in ASEAN countries that in turn has undermined the region’s efforts to combat the COVID-19 pandemic.

This situation continues to play out in the context of active efforts to vaccinate individuals in the region against dengue. Dengvaxia is the only commercially available vaccine that produces immunity against all 4 dengue serotypes. It is a live, attenuated vaccine that is only recommended for individuals who have previously been infected with dengue. This limits the effectiveness of the vaccine as it increases the risk of severe dengue in individuals who have not previously been infected [3]. Dengvaxia was introduced in 2016 in the Philippines, Indonesia, Thailand, and Singapore. It is costly, particularly in Indonesia, where the cost of the 3 recommended doses in 2016 was approximately US$207. In 2017, Dengvaxia caused a controversy in the Philippines after more than 733,000 children and 50,000 adult volunteers received the vaccine regardless of their serostatus. TAK-003 or DENVax is another potential vaccine against dengue that was developed by Mahidol University for which phase I and II trials were conducted in Singapore and Thailand. So far, it has produced sustained antibody responses against all 4 virus strains regardless of previous dengue exposure. Unlike vaccines for immunizing individuals against COVID-19, dengue vaccines were developed over a very long period of time [3,6].

## CHALLENGES AND IMPLICATIONS

Both the outbreak of dengue and the COVID-19 pandemic have led to a double-burden on ASEAN countries, as the COVID-19 pandemic has had a substantial impact on every aspect of life in these countries [6]. A concurrent surge in the rate of dengue infection within the region has also been observed in the course of the COVID-19 pandemic. While multiple factors may be responsible for the increase in dengue cases, one of the major factors that led to the surge was the disruption in the pathogen-host-vector relationship resulting from behavioural changes among the general population in response to the COVID-19 pandemic [5]. Several factors have played a role in this phenomenon, which are mainly caused by government-imposed social distancing and lockdown policies for controlling the spread of COVID-19 by decreasing viral transmission resulting from human contact [7]. However, these policies have led to considerable changes in the movements of individuals and have significantly increased individual contact with dengue vectors in vector-based transmission. For example, working from home increases the amount of time individuals spend in their local neighbourhoods. Increases in time spent in local residential areas raises the risk of human-vector contact despite the vector density in each respective area [8]. This trend was seen in Thailand, where around 2,000 new dengue cases were observed each month after the implementation of social distancing policies. Moreover, urban residential areas with a high concentration of low-rise buildings and denser drainage networks are ideal breeding habitats for dengue vectors [7].

Lockdown policies have also had an impact on dengue prevention programs. Most dengue control and surveillance measures, such as fogging, frequent local inspection of residential areas, source reduction, and peridomestic residual spraying, depend on human contact. Lockdown policies have limited the movements and coverage of dengue prevention programs, mainly due to the need for close contact between vector control teams and residents in affected areas. Limitations to practicing the full range of vector control measures in turn reduce the overall effectiveness of dengue prevention programs, thus leading to rapid increases in dengue cases [8].

Other impacts of the COVID-19 pandemic include fear and misinformation among the general population. Fear and anxiety has increased significantly across the general population in response to the COVID-19 pandemic, with many individuals fearing any medical management [9]. This phenomenon has led to a 30% to 40% decrease in hospital visits in the region, thus delaying critical medical assistance needed to diagnosis and manage many diseases [9-11]. With regard to dengue management, such delays have led to higher rates of severe dengue cases as well as morbidity and mortality related to new dengue cases [8].

Both diseases are known to have similar clinical indicators and symptoms, such as fever and malaise, which can potentially influence the management of both conditions due to misdiagnosis [8]. There can be delays in diagnosis, and disease management may be compromised due to similarities in the clinical courses of the conditions. The trajectory of disease management also influences the choice of diagnostic tools which impacts patient management. Several instances of cross-reactivity in response to rapid COVID-19 tests have been observed, indicating the possibility of co-infection or false positivity due to dengue [12]. Misdiagnosis of infection with either COVID-19 or dengue has been found to not only interfere with necessary and urgent management measures, but also can lead to a poor prognosis or even increase the risk of mortality. Increases in both dengue and COVID-19 infections have led to increases in the instance of various other co-infections. Several case reports from Indonesia and Thailand have shown misdiagnosis or delayed diagnosis, not just due to false-positive or false-negative interpretation, but also due to stigma prevalent among physicians themselves when diagnosing COVID-19 [13-16]. Misdiagnosis of COVID-19 as dengue also undermines the need for isolation between patients, leading to a higher risk of airborne transmission of COVID-19 to other patients. Likewise, misdiagnosis of dengue as COVID-19 undermines timely hydration which can lead to preventable death related to dengue [8].

The burden of both infections has also impacted the economies of the ASEAN member countries, particularly those with a high proportion of low-income and middle-income individuals. Dengue has caused a considerable burden in the region, with an annual cost per capita of approximately US$1.65 and approximately 372 disability-adjusted life years per million inhabitants [17]. The burden of both diseases may also affect epidemiological analysis and lead to underestimation of the number of cases reported. While the situation seems grave, there have also been positive developments. A unique report from Brazil showed possible cross-reactivity, indicating that cross-immunity from both dengue and COVID-19 can occur in patients, thus potentially leading to fewer COVID-19 infections [18].

Vaccination also poses a challenge to controlling the COVID-19 pandemic. COVID-19 vaccination rates in ASEAN countries vary and range from a less than 5% rate of complete coverage to more than 70%. For example, as of August 6, 2021, Singapore has immunized 74% of its population. Cambodia and Malaysia have the second-highest rate of inoculation at 47%, followed by Brunei and Thailand, with vaccination rates of 32% and 22%, respectively. Laos and Indonesia are next, each with an 18% vaccination rate, and the Philippines and Vietnam follow, with 11% and 7% vaccination rates, respectively [19]. Due to a lack of an updated vaccination report and the recent military coup that took place, Myanmar has the lowest reported vaccination rate, with only 3% of its population inoculated as of July 5, 2021 [19,20].

While acceptance of the COVID-19 vaccine is considerably high, with around a 93.3% acceptance rate for this 95% effective vaccine, and healthcare workers have a particularly high-level of acceptance of the vaccine, it is nonetheless the case that COVID-19 vaccine hesitancy due to various factors, including religious beliefs and anti-vaccine conspiracies, is still common and poses a challenge for vaccine advocates [21,22]. Past incidents of adverse effects following immunization, such as the Dengvaxia situation in which the risk of severe dengue increased among the vaccinated, have led to public mistrust concerning government vaccine programs in the Philippines. Surveys conducted after the controversy found that public confidence in vaccines the Philippines tanked from 93% in 2015 to 32% in 2018 [23]. Hesitancy has led to significant variation in the vaccine acceptance rates found in ASEAN countries. In countries with a lower rate of acceptance, hesitancy hinders the control of both dengue and COVID-19.

## RECOMMENDATIONS

While it is important for public health officials and healthcare facilities to divert their attention to the COVID-19 pandemic, it is also of the utmost importance to maintain awareness of vector control during the pandemic in response to the rising number of dengue infections. ASEAN countries should adapt dengue control programs in response to social distancing measures and other policy guidelines to address the COVID-19 pandemic. The governments of ASEAN countries should also prioritize digitizing their dengue surveillance systems. Mobile-based dengue surveillance systems have been developed for 2 cities in the Philippines and Indonesia and have shown promising results [24,25]. These systems allow healthcare workers to rapidly notify local and national healthcare authorities for every confirmed dengue case. The governments of ASEAN countries should also consider digitizing house-to-house inspections, which are currently limited due to social distancing practices. Larvae inspection, for example, can be conducted by requiring residents of high-risk areas to send photos of bodies of water near their homes.

Adequate protective gear and guidelines for vector control teams are essential for properly addressing the risk of COVID-19 infection and ensuring the safety of the population. Social mobilization against these diseases should also be strengthened using digital communication tools and social media platforms to spread awareness about COVID-19. Education on dengue prevention during the lockdown, beginning with information about the mosquito life cycle and covering breeding sites within potential dengue vectors, should be provided in order to gain the support of local residents toward vector control measures, especially in densely populated residential areas [7,8]. Dengue vector control teams should also pay close attention to the maintenance of buildings and other infrastructural elements to ensure effective vector control measures. In addition, management of both dengue and COVID-19 should be practiced in order to prevent misdiagnosis and delayed treatment.

The need for a critical triage algorithm to differentiate the 2 conditions is essential for implementing adequate management and treatment procedures, as well as to lower the number of preventable deaths from both dengue and COVID-19 [8]. Lastly, dengue should be closely monitored for the purposes of accurate and precise epidemiological reporting [7,8]. Further research is also needed to gain a better understanding of the relationship between the 2 diseases, particularly with regard to the cross-reactivity phenomenon. Vaccination for both diseases should be enforced and properly administered with transparency and according to objective policies to increase public acceptance of both vaccines, though further research is needed to determine any potential interactions between the vaccines [26].

## CONCLUSION

The COVID-19 pandemic has become the major public health focus of ASEAN countries and their healthcare systems. However, dengue infection still poses a danger to the populations of ASEAN countries, and the rate of dengue has continuously increased during the pandemic. Several countries in the region have already reported an increased number of dengue cases, indicating that a response is needed to mitigate the dangers of both dengue and COVID-19. Timely and appropriate guidelines and vector control programs are necessary for properly addressing the situation, not only to lower the number of cases, but also to ease the burden of dengue and COVID-19 management. Given the high rate of dengue infections in the region and the region’s contribution to the global dengue burden, it is imperative not only to continue, but also to strengthen efforts to address dengue, even during the COVID-19 pandemic. ASEAN countries should thus make a collective effort to address the high prevalence rate of dengue during the COVID-19 pandemic.

### Ethics statement

Ethic statement for the article is not needed due to its nature of perspective or review article.
